# Supplementary material for: Complex‐centric proteome profiling by SEC‐SWATH‐MS
Source: Mol Syst Biol. 2019 Jan 14;15(1):e8438. doi: 10.15252/msb.20188438 (PMC6346213; doi:10.15252/msb.20188438)
Supplement: Supplementary file 6 — Dataset EV5 [file MSB-15-e8438-s006.zip › feature_plots_corum/3064-2.pdf]

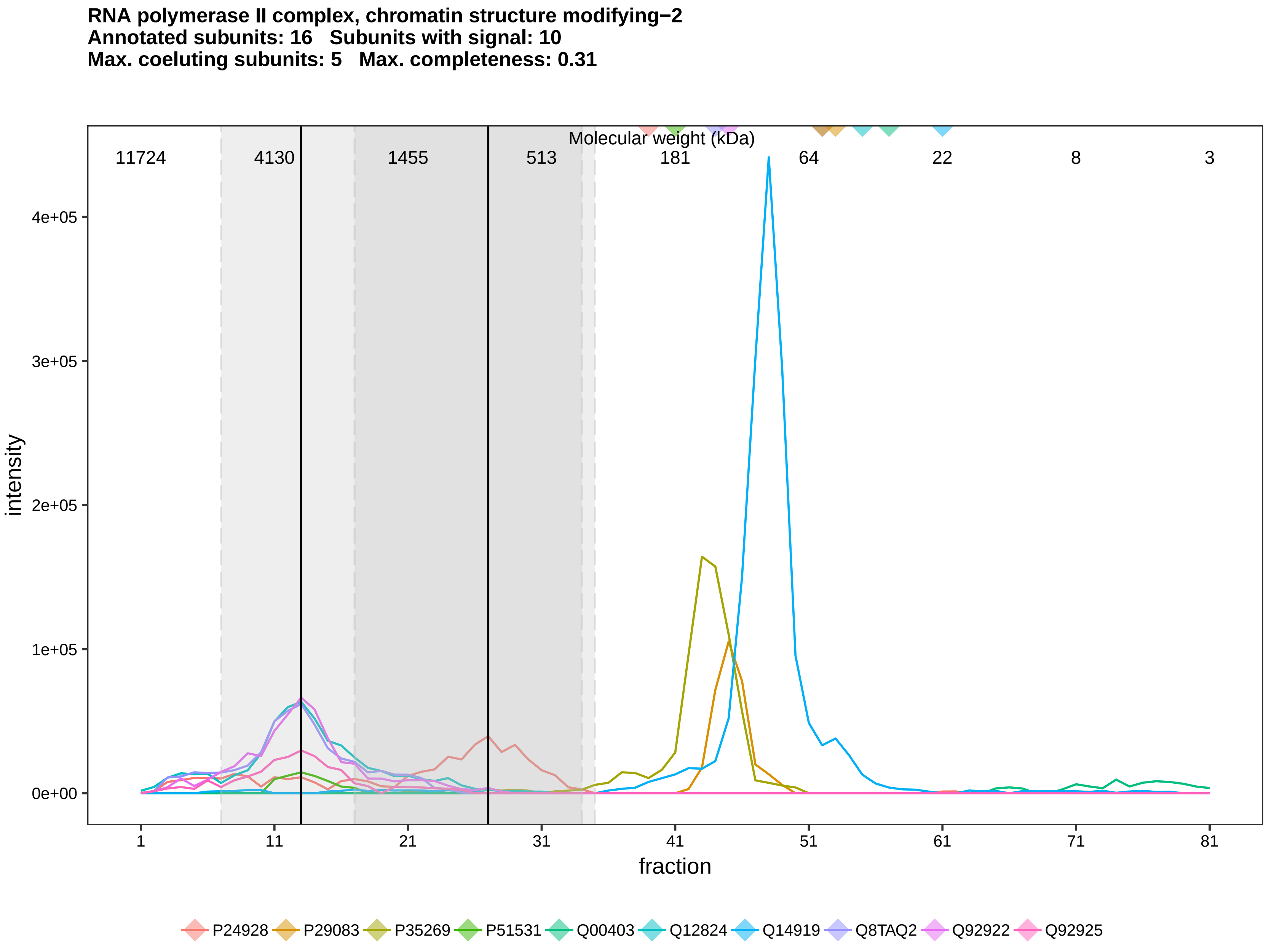

RNA polymerase II complex, chromatin structure modifying−2
Annotated subunits: 16 Subunits with signal: 10
Max. coeluting subunits: 5 Max. completeness: 0.31
Molecular weight (kDa)
11724
4130
1455
513
181
64
22
8
3
4e+05
3e+05
2e+05
1e+05
0e+00
intensity
1
11
21
31
41
51
61
71
81
fraction
P24928
P29083
P35269
P51531
Q00403
Q12824
Q14919
Q8TAQ2
Q92922
Q92925
